# Clinical outcome measures in juvenile idiopathic arthritis

**DOI:** 10.1186/s12969-016-0085-5

**Published:** 2016-04-18

**Authors:** Alessandro Consolaro, Gabriella Giancane, Benedetta Schiappapietra, Sergio Davì, Serena Calandra, Stefano Lanni, Angelo Ravelli

**Affiliations:** Istituto Giannina Gaslini, Genova, Italy; Università degli Studi di Genova, Genova, Italy

**Keywords:** Juvenile idiopathic arthritis, Outcome

## Abstract

Juvenile idiopathic arthritis (JIA), as a chronic condition, is associated with significant disease- and treatment-related morbidity, thus impacting children’s quality of life. In order to optimize JIA management, the paediatric rheumatologist has begun to regularly use measurements of disease activity developed, validated and endorsed by international paediatric rheumatology professional societies in an effort to monitor the disease course over time and assess the efficacy of therapeutic interventions in JIA patients.

A literature review was performed to describe the main outcome measures currently used in JIA patients to determine disease activity status.

The Juvenile Disease Activity Score (JADAS), in its different versions (classic JADAS, JADAS-CRP and cJADAS) and the validated definitions of disease activity and response to treatment represent an important tool for the assessment of clinically relevant changes in disease activity, leading more and more to a treat-to-target strategy, based on a tight and thorough control of the patient condition. Moreover, in recent years, increasing attention on the incorporation of patient-reported or parent-reported outcomes (PRCOs), when measuring the health state of patients with paediatric rheumatic diseases has emerged.

We think that the care of JIA patients cannot be possible without taking into account clinical outcome measures and, in this regard, further work is required.

## Background

Juvenile idiopathic arthritis (JIA) is a chronic disease characterized by prolonged synovial inflammation that may cause structural joint damage [1]. Nonreversible abnormalities may also occur in extra-articular organs, such as the eye (as a complication of iridocyclitis) or the kidney (due to systemic amyloidosis), or may result from adverse effects of drug therapies [2]. This morbidity may impair the quality of life of patients and their families [3].

The objectives of the management of JIA are to ameliorate patient symptoms and to improve inflammatory manifestations in an effort to improve health-related quality of life (HRQL) and prevent irreversible damage. The attainment of these goals is facilitated by the constant monitoring of disease course and child health status through the regular application of validated outcome measures [4, 5]. The incorporation of these assessments in daily care requires the use of simple and feasible tools, that are easily utilizable in a busy clinic.

In the past few years, numerous outcome measures have been developed and validated for use in children which JIA, including methods for scoring disease activity and disease damage, therapeutic response criteria, and questionnaires for the estimation of physical functioning and HRQL (reviewed in [6, 7]). Most recently, there has been an increased focus on parent- and child-reported outcomes (PCROs) [6, 8–10]. These instruments are considered valuable as they capture the parent and child perception of disease state and results of treatment. It is now agreed that the inclusion of PCROs in clinical practice may lead to improve the quality of care [11].

This review is aimed to provide an update on current research in outcome assessment in JIA, with special focus on physician centered disease activity measures.

### Disease activity

The regular measurement of disease activity level is essential to monitor the disease course over time in children with JIA and allows the assessment of the efficacy of therapeutic interventions [12, 13]. However, it is widely agreed that no single measure can reliably capture overall disease activity in all JIA phenotypes. Therefore, the use of the so-called composite disease activity scores has markedly increased in the last decade. These tools are made up by pooling several individual measures in a single instrument and have the advantage of integrating multiple aspects of the disease into one summary number on a continuous scale [14]. Composite scores are ideally suited for the assessment of disease activity in single patients, therapeutic efficacy in clinical trials, and to compare disease status between patients or patient groups.

### The juvenile arthritis disease activity score

In 2009, our group developed the first composite disease activity score for JIA, named Juvenile Arthritis Disease Activity Score (JADAS) [15]. The JADAS includes the following four measures: physician’s global assessment of disease activity, measured on a 0–10 visual analog scale (VAS) where 0 = no activity and 10 = maximum activity; parent global assessment of well-being, measured on a 0-10 VAS where 0 = very well and 10 = very poor; the erythrocyte sedimentation rate (ESR), normalized to a 0 to 10 scale; and a count of joints with active disease (Table 1).

**Table 1:** Construct and score range of the different available versions of the Juvenile Arthritis Disease Activity Score (JADAS)

	Physician global assessment	Parent/patient global assessment	Active joint count	Acute-phase reactant (range)	Score range (total)
JADAS					
• JADAS10	0–10-cm VAS	0–10-cm VAS	Simple count, 0–10^a^	Normalized ESR (0–10)^c^	0–40
• JADAS27	0–10-cm VAS	0–10-cm VAS	Reduced count, 0–27	Normalized ESR (0–10)^c^	0–57
• JADAS71	0–10-cm VAS	0–10-cm VAS	Simple count, 0–71	Normalized ESR (0–10)^c^	0–101
JADAS-CRP					
• JADAS10-CRP	0–10-cm VAS	0–10-cm VAS	Simple count, 0–10^a^	Normalized CRP (0–10)^b^	0–40
• JADAS27-CRP	0–10-cm VAS	0–10-cm VAS	Reduced count, 0–27	Normalized CRP (0–10)^b^	0–57
• JADAS71-CRP	0–10-cm VAS	0–10-cm VAS	Simple count, 0–71	Normalized CRP (0–10)^b^	0–101
cJADAS					
• cJADAS-10	0–10-cm VAS	0–10-cm VAS	Simple count, 0–10^a^	–	0–30
• cJADAS-27	0–10-cm VAS	0–10-cm VAS	Reduced count, 0–27	–	0–47
• cJADAS-71	0–10-cm VAS	0–10-cm VAS	Simple count, 0–71	–	0–91

^a^With a cutoff at ten, i.e., any active joint count higher than ten is given ten points

^b^The C-reactive protein CRP was truncated to a 0–10 scale according to the following formula: (CRP (mg/l) − 10)/10, similar to the normalized ESR used in the original JADAS. CRP values <10 mg/l are given 0 points and CRP values >110 mg/l are given ten points

^c^The ESR value is normalized to a 0–10 scale according to the following formula: (ESR (mm/h) – 20)/10. ESR values < 20 mm/h are given 0 points and ESR values > 120 m/h are given ten points

In the process of developing the JADAS, it was felt that the score components should have been selected among the six variables included in the ACR Pediatric core set [16]. However, two of the six variables included in the core set, namely restricted joint count and physical function assessment, were not included in the JADAS because they were considered to be relevantly affected by functional or structural damage [17]. It was deemed important to include parent global assessment, in order to incorporate parents’ perception of disease activity, even though this measure was also found to reflect functional damage, particularly in the later stages of illness [17].

Parent global assessment of well-being and physician’s global assessment of disease activity can be both assessed on a linear 10 cm VAS and in a 21-circle VAS [18].

Three versions of the JADAS were developed, each one differing in the active joint count incorporated: JADAS10, JADAS27 and JADAS71. The JADAS10 is based on the count of any involved joint, irrespective of its type, up to a maximum of ten joints: any joint count higher than ten yields ten points in the score. The JADAS27 includes a selected count of the following joints: cervical spine, elbows, wrists, metacarpophalangeal joints (from first to third), proximal interphalangeal joints, hips, knees and ankles. This is based on previous analysis that showed that the 27-joint reduced count is a good surrogate for the whole joint count in JIA [19].

The active joint count included in the JADAS71 was meant to be unrestricted. However, this version of the score was developed and validated using a rheumatologic examination form that included 71 joints [19]. It is important to remark that this form does not include the thoracic and lumbar spine, currently included in the standard rheumatologic examination adopted by the Paediatric Rheumatology International Trials Organization (PRINTO) and by the Pediatric Rheumatology Collaborative Study Group (PRCSG). To solve this formal incongruence, in clinical trials it is advised to combine cervical, thoracic and lumbar spine in a single joint. Notably, the sacro-iliac joints are not included in the active joint count since they cannot be clinically swollen nor do they present a limited range of motion.

The JADAS is calculated as the simple sum of the scores of its four components, which yields a global score of 0–40 for the JADAS10, 0–57 for the JADAS27, and 0–101 for JADAS71. The instrument is feasible and possesses both face and content validity. Furthermore, in the validation analysis, it has exhibited good construct validity, discriminant validity, and responsiveness to clinically important changes in a large patients’ dataset.

### The JADAS-CRP

Recently, Nordal et al. developed and validated an alternative version of the JADAS by substituting the ESR with the CRP [20]. JADAS-CRP was calculated similarly to the original JADAS as the simple sum of its four components, yielding a global score of 0–40, 0–57 and 0–101 depending on the joint count used for the JADAS10-CRP, JADAS27-CRP and JADAS71-CRP, respectively. JADAS-CRP versions were validated by showing their high correlation (*r* = 0.99) with the corresponding version of the original JADAS.

### The cJADAS

Recently, McErlane et al. have tested a clinical three-item version of the score, which excluded the ESR [21]. The modified score was named JADAS3-10, -27, -71, according to the active joint count included. However, we have suggested that the acronym cJADAS, i.e., clinical JADAS, is preferable in order to avoid any confusion due to the proximity of numbers [22]. The idea of developing a clinical version of the score was based on the notion that ESR and CRP are not routinely measured in all clinical settings, and therefore the JADAS versions including an acute phase reactant could be less feasible. The cJADAS correlated well with the original JADAS.

### The juvenile spondyloarthritis disease activity index

In 2014, Weiss et al. developed and validated the Juvenile Spondyloarthritis Disease Activity Index (JSpADA), a composite disease activity index for children and adolescents with juvenile spondyloarthropathy [23]. The index development was based on a modified Delphi consensus survey among a group of 106 international experts, who were asked to evaluate and rank a series of clinical and laboratory features as well as parent/patient-reported and physician-centered outcome measures. Items with a minimum 80 % consensus among raters were retained in the score. The following ten items were finally selected: arthritis, enthesitis, patient pain rating, acute phase reactants, morning stiffness, clinical sacroiliitis, uveitis, and back mobility. The final version of the JSpADA consists of eight items, each of which is given the same importance (value = 1). The range of possible score is, then, 0–8, with higher scores indicating greater disease activity.

Validation was carried out in a retrospective multicenter cohort of 178 children with newly diagnosed juvenile spondyloarthritis followed longitudinally. Three key domains that explained 58 % of the variance were identified by factor analysis: peripheral disease, axial disease, and uveitis. The instrument demonstrated moderate-to-high correlation with other established outcome measures, distinguished well between patients with active and inactive disease, and proved responsiveness to change in disease activity over time. Overall, the tool was found to be quick and easy to complete.

### Disease activity states and response to treatment

#### Inactive disease, low and high disease activity according to the JADAS

The composite scores are perfectly designed to follow over time the disease course of a child with JIA. However, the utility of these tools is greatly enhanced by the availability of criteria for identifying high and low levels of activity [24]. Table 2 shows cutoff values in the JADAS that correspond to the states of inactive disease, low disease activity, moderate disease activity and high disease activity. Cutoffs were developed for all JIA subtypes together and for oligoarticular and polyarticular JIA separately. Cutoffs for the parent/child acceptable symptom state were also developed, based on the subjective rating of the parent or the patients, reported in the Juvenile Arthritis Multidimensional Assessment Report (JAMAR) [25]. The cut-points were developed for all the original JADAS versions [26, 27] and for the cJADAS10 [22]. In the original studies, the term “minimal disease activity” was adopted; however, for sake of uniformity with adult rheumatology nomenclature, it was substituted with “low disease activity”. It is important to note that patients with systemic JIA and active systemic features were excluded from those studies, and disease activity cutoffs for this JIA category are still to be developed.

**Table 2:** Cutoffs for disease activity states in original and clinical JADAS versions

	JADAS10/71	JADAS27	cJADAS10
Oligoarthritis			
Inactive disease	≤1	≤1	≤1
Low disease activity	1.1 – 2	1.1 – 2	1.1 – 1.5
Moderate disease activity	2.1 – 4.2	2.1 – 4.2	1.51 – 4
High disease activity	>4.2	>4.2	>4
Polyarthritis			
Inactive disease	≤1	≤1	≤1
Low disease activity	1.1 – 3.8	1.1 – 3.8	1.1 – 2.5
Moderate disease activity	3.9 – 10.5	3.9 – 8.5	2.51 – 8.5
High disease activity	>10.5	>8.5	>8.5

#### Definition of inactive disease, remission, and minimal disease activity

A different approach to define the different disease activity states in JIA is based on the use of a core set of multiple criteria. With this approach, Wallace and co-workers in 2004 developed the preliminary criteria for disease remission in JIA through an international collaborative effort [28]. Based on these criteria, a patient is classified as having inactive disease when he/she has no joints with active disease, no systemic manifestations attributable to JIA, no active uveitis, normal values of acute phase reactants and a physician global assessment of disease activity indicating no disease activity. When the criteria for inactive disease are met for a minimum of six consecutive months while the patient is receiving anti-rheumatic medications, the patient is classified as being in the state of clinical remission with medication. When the criteria for inactive disease are met for a minimum of 12 consecutive months after the patient has discontinued all anti-rheumatic medications, the patient is classified as being in the state of clinical remission without medication. These criteria have recently been modified by providing a specific definition for uveitis and abnormal ESR and by including among the requirements for achieving the state of inactive disease the duration of morning stiffness of ≤15 min [29] (Table 3).

**Table 3:** Criteria for defining clinical inactive disease in oligoarticular (persistent and extended), polyarticular (RF+ and -), and systemic JIA*

Inactive disease
- No joints with active arthritis
- No fever, rash, serositis, splenomegaly, or generalized lymphadenopathy attributable to JIA
- No active uveitis as defined by the SUN Working Group [28]
- ESR or CRP level within normal limits in the laboratory where tested or, if elevated, not attributable to JIA
- Physician’s global assessment of disease activity score of best possible on the scale used
- Duration of morning stiffness of 15 min

*JIA* juvenile idiopathic arthritis, *RF* rheumatoid factor, *ESR* erythrocyte sedimentation rate, *CRP* C-reactive protein

*Wallace [29]

A more achievable goal for patients with JIA is the state of minimal disease activity. It was defined by Magni-Manzoni and co-workers in 2008 as the presence of a physician’s global rating of disease activity ≤3.4, a parent’s global rating of well-being ≤2.5 and a swollen joint count ≤1 in polyarthritis, and as a physician’s global assessment of disease activity ≤2.5 and a swollen joint count = 0 in oligoarthritis [30].

#### Measures of response to treatment

The most widely accepted criteria to define an improvement in patient disease course in response to a therapeutic intervention are the American College of Rheumatology (ACR) Pediatric response criteria developed in 1997 [16]. This criteria are based on the ACR core outcome variables for juvenile arthritis, namely physician global assessment of disease activity (10-cm VAS), parent/patient assessment of overall well-being (10-cm VAS), functional ability, number of joints with active arthritis (defined as joint effusion or limitation of motion accompanied by heat, pain, or tenderness), number of joints with limited ROM, and ESR. An ACR Pedi 30 response is defined as at least a 30 % improvement from baseline in three of six variables, with no more than one remaining variable worsening by >30 %. Similarly, the ACR Pedi 50, 70, 90, and 100 response definitions require 50 %, 70 %, 90 %, and 100 % improvement, respectively, in at least three core set variables without worsening of more than one variable by >30 %. Symmetrically, flare is defined as worsening of two variables by at least 40 % without improvement in more than one variable by 30 % [31]. Soon after their publication, these criteria became the gold standard for the assessment of response to therapy in JIA. The ACR Pedi 30 criteria are accepted by both the US Food and Drug Administration and the European Medicines Agency for all phase III trials in JIA seeking drug registration [32]. Recently, the ACR Pediatric 30 was adapted for use in clinical trials in systemic JIA, by adding, besides the six core set variables, the demonstration of the absence of spiking fever (>38 °C) during the week preceding the evaluation [33, 34].

Recently, Horneff and co-workers have presented a definition of improvement based on the JADAS10 [35]. The cutoffs for improvement are different according to the level of disease activity at baseline. The absolute changes of the JADAS10, that correspond to a meaningful improvement according to this definition, in patient being in low, moderate or high disease activity at baseline, are 4, 10, and 17, whereas the percent changes are 41 %, 53 % and 57 %, respectively. According to the validation analysis in the study, the performance of the ACR criteria was inferior to that of the JADAS10 improvement [35]. The ACR Pediatric response criteria highlight a change in disease state and are an important tool for assessment of clinically relevant improvement in disease activity. They are, therefore, ideally suited to be used in clinical trials that evaluate the efficacy of a new therapy in comparison to another therapy or a placebo. However, the nature of their calculation does not enable the measurement of patients’ actual disease activity at the beginning or end of a clinical trial or the comparison of one patient’s absolute response with that of another patient. Moreover, such a definition implies that achieving improvement or flare is dependent on the patient’s status at baseline, and therefore the absolute level of disease activity may be different for each patient even though they meet the same criteria for improvement or flare [36].

#### Toward a treat-to-target strategy

In the past 20 years the shift towards early aggressive interventions, the development of new therapeutic agents including methotrexate and biological medications and combination treatment strategies have radically changed the management of JIA patients and improved the long term outcome [37–39]. This progress has made the attainment of disease remission or, at least, minimal levels of disease activity, an achievable goal and the therapeutic aims and patients’ expectations were shifted towards an inactive disease status [40–42]. Complete disease quiescence is regarded as the ideal therapeutic target because its achievement was demonstrated to prevent further joint damage and disability, and may improve physical function and quality of life [43]. Both JADAS-based and definition-based criteria for inactive disease can be considered optimal target for a therapeutic intervention in JIA [44].

The current definition of inactive disease requires the total absence of any sign or symptom of disease activity and is, therefore, extremely strict. Even meeting the JADAS cutoffs for remission is challenging, being the required score ≤1 for all JADAS versions and for both oligoarthritis and polyarthritis. However, achievement of a real inactive disease either in everyday clinical practice or in pharmaceutical trials is still problematic in many patients, particularly those with polyarticular or systemic JIA. It has been proposed that in clinical care a more attainable goal could be to induce and maintain at least a state of minimal (or low) disease activity, which is an intermediate state between high disease activity and remission, though very close to remission [30]. In adult patients with rheumatoid athritis (RA), this state is deemed to be a useful target of treatment by both the physician and the patient, given current treatment possibility and limitations [45].

Studies in adult patients with (RA) have demonstrated that clinical and biological outcomes are improved if a treat to target strategy is implemented, i.e., through the practice to aim for minimal levels of disease activity by frequent adjustment of therapy according to quantitative indices [46–48]. Indeed, the strategy of tight control, aiming at remission, has been considered more important than the therapeutic agent [49]. A similar approach has not yet been reported for JIA; however, the achievement of the state of inactive disease, at least once in the first five years of the disease, was found to be associated with lower levels of long-term damage and lower functional impairment in children with polyarthritis [43]. In addition, a greater magnitude of clinical response in the first six months of methotrexate therapy was found to predict a more favorable long-term outcome [50].

### Parent/patient reported outcomes

In recent years, increasing attention has been paid to PCROs in JIA [8]. Incorporation of these measures in patient assessment is deemed important as they reflect the parents’ and children’s perception of the disease course and effectiveness of therapeutic interventions. Information obtained with parent or child centered measures may contribute significantly to medical decision-making and increase the probabilities of success in patient care. Indeed, since physician’s therapeutic decisions are of primary importance to parents and patients, integration of their perspective in clinical evaluation may facilitate concordance with physician’s choices and compliance with therapeutic prescriptions [11, 51, 52]. In the chronic phase of the disease, patients or parents might report high perceived disease activity, high pain levels, high fatigue and low health-related quality of life (HRQOL) irrespective of current active disease. These measures should lead to support the patient in his/her perceived impaired health status other than to the adjustment of medication (except in case of side effects) and could help the physician to recognize the patients at risk for impaired perceived health in its broadest way.

A wide variety of measures for the assessment of PCRO in children with JIA is currently available, ranging from VAS for rating of child’s overall well-being and intensity of pain, to questionnaires for the evaluation of functional ability and HRQOL [18, 53–59]. The issue of drug tolerance has also been recently addressed, through the development of a methotrexate intolerance score [60]. However, valuable insights into the influence of the disease and its treatment on the child and his family may be provided by other PCRO not addressed by conventional instruments, such as evaluation of morning stiffness and overall level of disease activity, rating of disease status and course, proxy- or self-assessment of joint involvement and extraarticular symptoms, and assessment of therapeutic compliance and satisfaction with the outcome of the illness. The JAMAR is a recently developed clinical tool that groups all the main PCROs for children with JIA [25]. The JAMAR provides the attending physician with a thorough and systematic overview of the patient status to be scanned briefly at the start of the visit. This approach facilitates the focus on issues that require attention, leading to more efficient and effective clinical care and including valuable insights into the influence of the disease and its treatment. However, the JAMAR, in spite of its multidimensional nature, does not explore some relevant domains, e.g., fatigue or the impact of the disease in work-related issues.

Currently, a clinical instrument that groups all PCROs used in the assessment of children with JIA does not exist.

The American National Institute of Health has created a cooperative network of researchers to develop new PCRO scales using modern measurement theory, the Patient-Reported Outcomes Measurement Information System (PROMIS) [61]. This initiative is aimed to replace the multiplicity of disease-specific scales with generic assessment tools that can be used across medical conditions, including juvenile arthritis [62]. The PROMIS measurement tools are systematically developed using both qualitative and psychometric methodologies, including item response theory, to create either static short forms or computerized adaptive versions that can be administered in an array of clinical and research settings [63]. Validated PROMIS measures are currently being investigated for use in patient chronic pain conditions including JIA [64].

Finally, composite disease activity scores for JIA entirely based on parent- or child reported outcome measures were recently developed [65]. The Juvenile Arthritis Parent Assessment Index (JAPAI) and the Juvenile Arthritis Child Assessment Index (JACAI) are composed of the following items: 1) parent/child rating of overall well-being on a 0–10 cm VAS (0 = best; 10 = worst); 2) parent/child rating of pain intensity on a 0–10 cm VAS (0 = no pain; 10 = very severe pain); 3) assessment of physical function; 4) assessment of HRQL (excluded in a three-item version of the scores). Scores of physical function and HRQL tools included in the composite scores are converted to a 0–10 score by specific formulae, yielding a global score of 0–40 for the four-item versions and of 0–30 for the three-item versions. The JAPAI and the JACAI demonstrated to be valid instruments for the assessment of disease status in JIA and to monitor disease activity over time.

### Damage

Prolonged synovial inflammation characterizes the clinical picture of JIA: this, if not properly treated, may cause irreversible alterations in joint structures. The involvement of extraarticular organs/systems, such as the eye (as a complication of chronic anterior uveitis) or the kidney (due to systemic amyloidosis), may lead to permanent changes. Moreover, the side effects due to the prolonged administration of medications may also cause permanent or long-lasting damage [1].

Morbidity in JIA patients can be evaluated in terms of functional disability or by assessing structural joint damage through radiographs or other imaging modalities. However, these tools may not capture the multiple forms of damage that children with JIA may develop over time, such as micrognathia, height retardation, localized growth disturbances, pubertal delay, or visceral organ failure [66]. The Juvenile Arthritis Damage Index (JADI) is a clinical tool that enables a thorough detection of articular and extra-articular damage in children with JIA [2], through a simple, easy and quick score which takes just 5–15 min in the daily clinical setting. The information obtained by physical examination and from the patient’s clinical history is sufficient to complete the questionnaire and no radiographic examinations are required. The JADI is aimed to capture damage, defined as persistent changes in anatomy, physiology, pathology or function, present for at least six months, which may be the consequence of previous active disease, side effects of therapy, or comorbid conditions, and is not due to currently active arthritis. The index is composed of two parts, one devoted to the assessment of articular damage (JADI-A) and one devoted to the assessment of extra-articular damage (JADI-E). The maximum total score is 72 for JADI-A and 17 for JADI-E. Damage is often irreversible and cumulative and, thus, damage scores are expected to remain stable over time or to increase. However, because some forms of damage may improve or even resolve in growing children, in some cases the total score may decrease.

## Conclusions

In the past decade, an intense research effort has been made to design and validate new outcome measures for the clinical assessment of children with JIA. The proposed instruments have expanded the health domains that can be evaluated quantitatively. This progress may promote the regular administration of parent/child questionnaires in standard clinical care. Although the use of standardized outcome measure may be facilitated in tertiary care settings, with larger staff assistance, most of the described measures have been specifically designed for regular administration in a busy clinical setting, with particular attention to feasibility and acceptability in daily care. Patient or parent questionnaires can be completed in the waiting area before the patient is called into an examining room and the physician should spend only a few seconds reviewing and scoring the data.

Furthermore, the use of quantitative measures will increase the reliability of comparison of patient populations and the analysis of the effectiveness of current and novel therapeutic protocols. To achieve these goals, the available tools or those that will be developed in the future should be tested in patients seen in different geographic areas and embraced by pediatric rheumatologists practicing in diverse countries and continents. A potential caveat is that most outcome measures described in this review have been developed and validated for use in children and adolescents. To foster harmonization with the assessments made in young adults in the context of transition of care, there is a need to adapt the new instruments for use in the adult age and to scrutinize their correlation with the existing adult-oriented tools.
